# PNI Biomarkers and Health Outcomes in College Women

**DOI:** 10.3390/healthcare2020207

**Published:** 2014-04-28

**Authors:** Shih-Yu Lee, Mugdha Vasireddi, Yu Ping Chen, Yong Tai Wang, Julia Hilliard

**Affiliations:** Byrdine F. Lewis School of Nursing and Health Professions, Department of Biology, Georgia State University, Atlanta, GA 30302, USA; E-Mails: mvasireddi1@gsu.edu (M.V.); ypchen@gsu.edu (Y.P.C.); ywang2@gsu.edu (Y.T.W.); jhilliard@gsu.edu (J.H.)

**Keywords:** sleep, stress, depression, PNI biomarkers, college women, health outcomes

## Abstract

Sleep disturbance has been found to trigger a stress response with a subsequent activation of the psychoneuroimmunological (PNI) pathway associated with adverse health outcomes. This study aimed to assess the association among selected PNI biomarkers, sleep disturbances, and adverse health outcomes (depressive symptoms, physical symptoms). A stratified, quota sample (14 poor sleepers and 15 good sleepers) was drawn from a pool of healthy college women from a larger scale of study. The participants reported their sleep, stress, depressive, and physical symptoms. Wrist actigraphy was used to collect objective sleep data, and the Enzyme-Linked ImmunoSorbent Assay was used to assess PNI biomarkers. Poor sleep quality, higher stress perception, elevated serum serotonin, and lower serum interleukin-10 explained 75.3% of the variances for the depressive symptoms. Poor sleep quality along with delayed peak activity rhythms accounted 31.4% of the physical symptoms. High serotonin and tumor necrosis factor-α were the significant predictors for poor sleep efficiency, and serotonin was the single significant predictor for poor daytime functioning. Stress and sleep disturbances negatively impact the health of college women and should be as part of regular check-ups on campus. PNI effects on health outcomes should be further explored. Educational materials in the areas of sleep hygiene, health impacts from sleep disturbances, and strategies to maintain synchronized circadian rhythms should be mandatorily included in the college curriculum.

## 1. Introduction

Sleep disturbances are more prevalent among American females than males, according to the 2007 Sleep in America Poll survey that found about half of the women in the United States experienced sleep problems nightly [1]. Sleep disturbances have been found to trigger a stress response with subsequent activation of the psychoneuroimmunological (PNI) pathway that has an impact on fatigue, depression, and adverse health outcomes [2,3]. The pro-inflammatory cytokines, such as interleukin (IL)-1, IL-6, and tumor necrosis factor (TNF)*-*α*,* increase slow wave sleep; and the anti-inflammatory cytokines, such as IL-4 and IL-10, inhibit sleep [4]. Thus, studies of the association between sleep and health outcomes should also consider PNI markers as the predictor.

Sleep disturbances are prevalent in the U.S.; however, many Americans do not take actions that address their sleep problems [5]. It has been found that about 45% of adolescents do not sleep enough [6]; studies of college students have found that more than 60% of them were poor sleepers [7,8]. Compared to men, women are more vulnerable to the effects of sleep disturbances because of cycling hormones and engagement in multiple social roles [1,9]. Although there is emerging evidence of the relationships between sleep disturbances and adverse health outcomes [10], most of the findings have been derived from studies with a specific disease-based population or with healthy human beings but under an artificial context, such as monitoring sleep in the sleep lab. A study in a natural context and over an extended period of time may lead to a better understanding of the pathway between sleep disturbances and health outcomes.

Little is known about the associations among PNI biomarkers, sleep disturbances, and adverse health outcomes in healthy adult women. Without prospective data, gaps remain in our understanding of the mechanisms underlying how sleep disturbances earlier in life may contribute to future health problems. Better understanding of these mechanisms may lead to tailored interventions to prevent or delay health problems. In this prospective, stratified quota, comparative study, we intend to lay the foundation for a developmental model of the role of sleep in several key mechanisms responsible for adverse health-related wellbeing (depressive symptoms, physical symptoms) and PNI markers in female adults.

The biomarkers selected for this study are based on PNI effects resulting from sleep disturbances as reported in prior studies [3,11], and include: (1) Endocrine axis: cortisol, melatonin, serotonin; (2) Immune axis: pro-inflammatory, such as IL-1β, IL-6, TNF-α, anti-inflammatory cytokines (*i.e.*, IL-10), and C-reactive protein (CRP). For example, cortisol is usually referred to as the “stress hormone” as it is involved in the response to stress—it increases blood pressure and blood sugar, and disturbs sleep [12]. CRP responds to pro-inflammatory cytokines, particularly IL-6 and is considered a sensitive marker of low-grade inflammation [13]. Studies have demonstrated an inverse correlation between CRP concentration and cardiovascular disease in both type 1 and type 2 diabetes [14]. The specific aim of this study was to assess the associations among selected PNI biomarkers, sleep disturbances, and adverse health outcomes (morning fatigue, depressive symptoms, and physical symptoms). The following research questions were addressed:

1. Are there any differences in the characteristics of perceived stress, morning fatigue severity, depressive symptoms, daytime sleepiness, nocturnal total sleep time, activity level, physical symptoms, and PNI markers between the good sleepers and poor sleepers?
2. To what degree are depressive symptoms explained by the individual’s personal characteristics (*i.e.*, age, body mass index), perceived stress, sleep disturbances, and PNI markers?
3. To what degree are physical symptoms explained by an individual’s personal characteristics (*i.e.*, age, body mass index), perceived stress, sleep disturbances, and PNI markers?

## 2. Methods

### 2.1. Study Design and Participants

The Impaired Sleep Model [15] was used to guide this correlational exploratory study. Approval for this study was obtained from the University Institutional Review Board in the southern United States. Informed consent was obtained from each study participant. The participants (15 poor sleepers and 14 good sleepers) in the current 3-day study were a cohort from a larger scale study, which was a 7-day, prospective, naturalistic observation study [8]. Details of the larger scale study has been reported elsewhere [8]; in brief, the study participants were college women at least 18 years of age and self-reported without any existing physical and mental disorders. Sleep strata (see Table 1), determined by the Pittsburg Sleep Quality Index (PSQI) [16] and Sleep Deviation Index (SDI) [17], was used to classify poor or good sleepers. The SDI is an absolute value of the ratio of the difference between actual total sleep time and preferred sleep time to 24 hours. The poor sleeper was operationally defined as occurring when an individual scored more than five on the PSQI and in the highest quintile in the SDI in both school and non-school nights. The SDI ranges from 0 to 1; a deviation index of 0 reflects a perfect match between the amount of sleep that is needed and the amount of sleep actually acquired [17].

**Table 1:** Selection strata.

Sleep stratum	Phase I sleep quality index in the PSQI	Phase I school night sleep deviation index	Phase I non-school night sleep deviation index
Poor sleeper	>5	Highest quintile	Highest quintile
Good sleeper	≤5	Lowest quintile	Lowest quintile

### 2.2. Study Instruments

The following instruments were used for data collection in the current study. A structured demographic form was used for collecting demographic information. Sleep disturbances in the week immediately preceding the questionnaire were assessed by the General Sleep Disturbance Scale (GSDS), a 21-item, 0–7 rating scale; a score equal or above 3 indicates poor sleepers [18]. Sleep disturbances in the past month were evaluated by the Pittsburgh Sleep Quality Index (PSQI), a 19-item, 0–3 rating scale; a score above 5 suggests insomnia [16]. The PSQI includes 7-subscale: sleep quality, sleep latency, sleep duration, sleep efficiency, sleep disturbances, use of sleeping medication, and daytime dysfunction. Daytime sleepiness was measured by the Epworth Sleepiness Scale (ESS), an 8-item, 0–3 rating questionnaire; higher scores indicate a higher degree of sleepiness [19].

The Numerical Rating Scale for Fatigue (NRS-F) [20], a 7-item scale with a possible score range of 0–10 (highest severity), was used to assess morning fatigue. The NRS-F has been used in several studies with healthy adult women. The Center for Epidemiologic Studies Depression Scale (CESD) [21], a 20-item scale with a possible score range of 0–60, was used to measure the study participants’ depressive symptoms. Study participants with CESD scores ≥16 were referred to their healthcare provider for further assessment. The Physical Symptom Inventory (PSI) [22], an 18-item scale with two subscales (symptoms (e.g., headache, infection) and symptoms-related doctor visit) was used to measure participants’ physical health. The possible score range is 0–18 for each subscale. The perceived stress measured by the Perceived Stress Scale (PSS) [23], a 10-item scale with a possible score range of 0 to 40, was used to assess the stress level results from daily life. All of these instruments have established reliability and validity, and have been used in our previous studies, with the exception of the PSI; however, it has been used in several studies with college students.

In addition to the self-reported questionnaires, we also included objective measurements in this study: wrist actigraph and PNI biomarkers. A wrist actigraph is a light-weight, watch-like motion sensor monitor (Mini Motionlogger Actigraphy, octagonal motionlogger, Ambulatory Monitoring Inc., Ardsley, NY, USA), which was used to measure total nocturnal and daytime sleep time, wake after sleep onset, and circadian activity rhythms. The assays kits (described later), which have excellent measurement properties, were used for evaluating the selected PNI-affected biomarkers by using a 7 mL blood sample.

### 2.3. Procedure

The questionnaires, along with wrist actigraphy data, and selected PNI biomarkers, were collected. Blood sample collection appointments at the Biology Department were coordinated by the first author to a written protocol and corresponded to the participants’ menstruation cycle (within 7 days after the menstruation period). In order to ensure valid measurements, the samples were obtained under the following standard conditions: (1) the participant sat in a comfortable position; (2) blood was drawn by a skillful phlebotomist by venipuncture through a 21-gauge needle with one attempt, the phlebotomist waited for at least 20 minutes prior to the second attempt if the first attempt was not successful; (3) the room was comfortably warm and quiet; (4) all fasting blood draws were done in the morning between 7:30 and 9:00 a.m.; and (5) a light breakfast, including milk or soy milk and nutrient bar, were provided after the blood draw. All study participants were asked to sit in the lab for a few minutes to ensure no adverse event (e.g., dizziness, uncomfortable feelings) occurred before they left the lab.

At the end of the 3-day study, all the participants received a sleep hygiene booklet [24], a report on her sleep patterns, and a report of any abnormal biomarkers for her to discuss with her primary health care provider. All the blood samples were labeled with an assigned code number and stored in a freezer (−80 °C) until assay. Only the first author had access to the code book.

### 2.4. Data Analysis

The data were generated in the following categories for each subject: (1) demographic data; (2) self-reported stress, sleep data, and morning fatigue; (3) two indices of adverse health outcomes (CESD, PSI); (4) selected PNI biomarkers; and (5) wrist actigraphy data. The wrist actigraphy data were analyzed using activity counts to determine sleep maintenance using Webster’s criteria [25]. The automatic sleep-scoring program (Action 4, software program, Ambulatory Monitoring Inc., Arbsley, NY, USA) was used to calculate TST (total sleep time) in minutes and WASO (wake after sleep onset) in percentage. The average of the 3-day TST and WASO were used for data analysis.

The biomarker analyses were performed in the lab at the Biology Department in the University using established laboratory protocols for specimen handling and analysis. Preparations of assay controls followed the instructions from the manufacturers. Inflammatory cytokines (IL-6, TNF-α, IL-1b, and IL-10), CRP, melatonin, serotonin, and cortisol were measured using the Enzyme-Linked ImmunoSorbent Assay (ELISA). Cytokines were measured sing “sandwich” ELISA kits purchased from BD Biosciences (San Jose, CA, USA). CRP levels in the serum were detected by using “sandwich” ELISA purchased from LDN, Germany. Serotonin and cortisol levels in the serum were detected using “competition” ELISA kits purchased from LDN, Germany. Melatonin levels in the serum were quantified using “competition” ELISA kits purchased from GenWay Biotech, Inc. (San Diego, CA, USA). A standard curve was plotted using the absorbance readings measured for standards against the known concentration of the standards. Non-linear regression, recommended by the manufacturers, was used to interpolate the protein concentration in the samples from the standard curve.

We entered data into Statistical Package for the Social Sciences (SPSS), version 18.0. Internal consistency reliability of the survey instruments was calculated as appropriate using Cronbach’s alpha coefficients. Descriptive statistics were used to describe the sample characteristics, PNI biomarkers, sleep variables, and adverse health outcomes. Chi-Square tests and ANOVAs were used to examine possible differences on demographic variables between the two groups (poor and good sleepers). Correlations, regression models, and t-tests were used to answer the research questions. 

## 3. Results

Based on the PSQI score and the SDI from the larger scale study, a total of 29 students with a mean age of 25.3 (SD = 7.1) who met the inclusion criteria as either good sleepers (*n* = 14) or poor sleepers (*n* = 15) were invited back for the current study (see Table 2). Independent t-tests showed there were statistically significant differences between good and poor sleepers on the PSQI global score (t [27] = −5.08, *p* < 0.001) and SDI in both full week (t [27] = −2.37, *p* < 0.05) and weekdays (t [27] = −3.14, *p* = 0.004).

### 3.1. Characteristics between Good Sleepers and Poor Sleepers

Table 3 presents descriptive statistics for perceived stress, daytime sleepiness, depressive symptoms, physical symptoms, nocturnal total sleep time, wake after sleep onset, circadian activity rhythms, morning fatigue severity, acrophase (peak activity level), and biomarker variables for the whole group in the current study and for the two subgroups (good and poor sleepers). Compared to the good sleepers the poor sleepers reported statistically significantly higher stress perception, depressive symptoms, daytime sleepiness, and more adverse physical symptoms. Compared to the good sleepers, the poor sleepers had higher levels of pro-inflammatory cytokines (IL1-β, IL-6, and TNF-α), anti-inflammatory cytokines (IL-10), cortisol, and serotonin levels; however, there were no statistically significant differences between the two groups in this small sample. On the other hand, compared to the good sleepers, CRP and melatonin levels were lower in the poor sleepers, though there were no statistically significant differences.

**Table 2:** Characteristics of the study participants.

Variables	Whole Group (*N* = 29)	Good Sleepers (*n* = 14)	Poor Sleepers (*n* = 15)
Age, years (Mean ± SD)	25.3 ± 7.1	24.9 ± 4.8	25.8 ± 8.9
Ethnic group (*n*[%])			
White	8 (27.6)	4 (28.6)	4 (26.7)
Black	9 (31.0)	3 (21.4)	6 (40.0)
Hispanic	4 (13.8)	4 (28.6)	---
Asian	8 (27.6)	3 (21.4)	5 (33.3)
Currently working (*n*[%])	15 (51.7)	9 (64.3)	6 (40.0)
Marital status (*n*[%])			
Married/partner	9 (31.0)	5 (35.7)	4 (26.6)
Single	19 (65.5)	9 (64.3)	10 (66.7)
Financial difficulty (*n*[%])	20 (69.0)	9 (64.3)	11 (73.3)
BMI (Mean ± SD)	24.4 (6.6)	25.4 (7.8)	23.4 (3.5)
SDI (Mean ± SE)			
7-days	0.054 ± 0.010	0.031 ± 0.007	0.075 ± 0.017 *
Weekdays	0.066 ± 0.011	0.034 ± 0.008	0.095 ± 0.017 **
Weekends	0.051 ± 0.009	0.034 ± 0.009	0.067 ± 0.015
PSQI (Mean ± SD)	6.27 ± 2.99	4.14 ± 4.1	8.3 ± 2.6 **

Note: * *p* < 0.05, ** *p* < 0.01. Abbreviations: BMI, Body Mass Index; SDI, Sleep Deviation Index; PSQI, Pittsburg Sleep Quality Index.

**Table 3:** Mean and standard deviation (mean ± SD) for the major variables.

Variables (cut-off)	Whole Group (*N* = 29)	Good Sleepers (*n* = 14)	Poor Sleepers (*n* = 15)
PSS (>13.6)	14.5 ± 6.5	11.4 ± 5.4 *	17.3 ± 6.3 *
ESS (>9)	8.3 ± 4.8	5.5 ± 3.7 **	11.0 ± 4.2 **
CESD (>16)	9.9 ± 7.3	6.5 ± 3.2 *	13.1 ± 8.7 *
PSI	5.1 ± 2.9	3.9 ± 2.3 *	6.3 ± 2.9 *
TST (<420 minutes)	397.8 ± 58.2	402.3 ± 41.8	393.6 ± 71.5
WASO (>10%)	5.5 ± 4.1	4.9 ± 3.6	6.0 ± 4.6
CAR	0.68 ± 0.11	0.70 ± 0.09	0.66 ±0.13
NRS-F (>3.2)	3.6 ± 1.5	3.4 ± 1.4	3.7 ± 1.5
Acrophase (time format)	15:43 ± 1:34	15:18 ± 1:16	16:09 ± 1:46
IL-1B (pg/mL)	0.57 ± 0.65	0.34 ± 0.34	0.72 ± 0.84
IL-6 (pg/mL)	4.16 ± 7.47	3.89 ±5.56	4.40 ± 9.1
IL-10 (pg/mL)	15.99 ± 20.10	10.05 ± 5.57	21.53 ± 26.67
TNF (pg/mL)	0.79 ± 1.50	0.49 ± 0.89	1.06 ± 1.89
CRP (<6 mg/L)	2.88 ± 3.21	3.59 ± 3.98	2.23 ± 2.23
Cortisol (µg/mL)	41.44 ± 3.04	40.90 ± 3.39	41.95 ± 2.70
Melatonin (pg/mL)	37.62 ± 25.75	43.15 ± 32.61	32.47 ± 16.74
Serotonin (ng/mL)	231.47 ± 321.05	162.82 ± 36.13	295.53 ± 442.52

Note: * *p* < 0.05, ** *p* < 0.01. Abbreviations: PSS, Perceived Stress Scale; ESS, Epworth Sleepiness Scale; CESD, Central Epidemiologic Scale-Depression; PSI, Physical Symptom Inventory; TST, total sleep time; WASO, wake after sleep onset; CAR, Circadian Activity Rhythms; NRS-F, Numerical Rating Scale for Fatigue; IL, interleukin; TNF, Tumor Necrosis Factor; CRP, C-reactive protein.

Table 4 presents the non-parametric correlational analyses between biomarkers and stress (PSS), sleep disturbances (PSQI), daytime sleepiness (ESS), depressive symptoms (CESD), and total physical symptoms (PSI). Only two markers, serotonin and IL-10, had a statistically significant associated with the stress perception and sleep-associated variables in the small sample. Serotonin was significantly associated with poor sleep quality and more physical symptoms. IL-10 was the only marker significantly associated with severity of daytime sleepiness. Participants’ perception of their overall sleep quality during the past month was associated with lower stress levels, less daytime sleepiness, and fewer depressive and physical symptoms (*p* < 0.05). Those who reported more physical symptoms also experienced more daytime sleepiness (*p* < 0.05).

**Table 4:** Correlations between stress, sleep, health-related well-being, and biomarkers (*n* = 29).

Variables	PSS	PSQI	ESS	CESD	PSI	IL1-*β*	IL-6	IL-10	TNF-α	Cortisol	CRP	Melatonin	Serotonin
**PSS**	---												
**PSQI**	0.44 *	---											
**ESS**	0.58 **	0.51 **	---										
**CESD**	0.71 **	0.57 **	0.35	---									
**PSI**	0.29	0.40 *	0.42 *	0.47 *	---								
**IL-1*β***	−0.01	0.05	0.04	−0.01	0.04	---							
**IL-6**	−0.01	0.09	−0.03	0.02	−0.16	0.09	---						
**IL-10**	0.28	0.17	0.42 *	0.02	0.09	0.17	0.32	---					
**TNF-α**	0.26	0.16	0.004	0.18	−0.16	0.26	0.09	0.18	---				
**Cortisol**	−0.05	0.28	0.19	0.10	0.22	0.11	0.04	0.25	0.10	---			
**CRP**	−0.24	0.09	−0.29	−0.13	−0.18	−0.24	0.64 **	0.10	−0.05	0.15	---		
**Melatonin**	0.05	−0.07	0.09	0.13	−0.03	−0.13	0.06	0.12	0.11	0.31	−0.04	---	
**Serotonin**	0.12	0.39 *	0.34	0.24	0.44 *	−0.02	0.31	0.28	−0.26	0.41 *	0.30	0.25	---

Note: * *p* < 0.05, ** *p* < 0.01. Abbreviations: PSS, Perceived Stress Scale; PSQI, Pittsburg Sleep Quality Index; ESS, Epworth Sleepiness Scale; CES-D, Center of Epidemiology Scale-Depression; PSI, Physical Symptom Inventory; IL, interleukin; TNF, Tumor Necrosis Factor; CRP, C-reactive protein.

### 3.2. Predictors for Depressive Symptoms and Physical Symptoms

Regression models were used to explore which variables (personal characteristics, perceived stress, sleep disturbances, and PNI markers) were statistically associated with depressive symptoms and physical symptoms. The confounding variables, age and BMI, were entered first, and neither was a significant predictor for depressive symptoms and physical symptoms. Therefore, age and BMI variables were removed from the model and the stepwise regression was conducted for the stress, sleep disturbances and PNI markers variables. The PSQI global score (t = 3.66, *p* = 0.001) along with higher serotonin levels (t = 3.49, *p* = 0.002), perceived higher stress (t = 3.95, *p* = 0.001), and lower IL-10 levels (t = −2.33, *p* = 0.029) explained 75.3% of the variances for the depressive symptoms which were measured by CESD (F [4,23] = 21.61, *p* < 0.001). The global PSQI (t = 2.42, *p* = 0.023) along with acrophase (t = 2.27, *p* = 0.032) accounted for 31.4% of the physical symptoms (F [2,25] = 7.18, *p* = 0.003), indicating the individuals with poor sleep quality and delayed peak activity rhythms (*i.e.*, evening person) also experienced more physical symptoms. Because sleep disturbances measured by the global PSQI were the significant predictor for depressive symptoms and physical symptoms, an exploratory analysis was conducted to examine if there is any association between the selected PNI biomarkers and PSQI, including both the global scale and its subscales. None of the biomarkers were the significant predictors for the global PSQI; however, serotonin (t = 3.94, *p* = 0.001) and TNF-α (t = 3.37, *p* = 0.002) are the significant predictors for poor sleep efficiency, and serotonin (t = 4.19, *p* < 0.001) was the single significant predictor for poor daytime functioning. 

## 4. Discussion

This study examined to what degree depressive symptoms and physical symptoms were explained by the selected personal characteristics (*i.e.*, age, body mass index), perceived stress, sleep disturbances, and PNI markers in a group of college women. In addition, we tested different characteristics of the PNI biomarkers between the good and poor sleepers. Data from this study showed college women are distressed, sleep deprived, sleepy during daytime, fatigued, and depressed. Poor sleep quality in the past month along with high serum serotonin levels, higher stress perception, and lower IL-10 predicted the severity of depressive symptoms. Poor sleep quality in the past month along with a delayed acrophase (peak activity level) predicted more physical symptoms. Data from this study showed that compared to the good sleepers the poor sleepers experienced significantly higher stress, more depressive symptoms, higher daytime sleepiness, and more adverse physical symptoms.

Elevated pro-inflammatory cytokines, such as IL-6 and TNF-α, along with CRP have been found in depressive patients [26]; however, the association between CRP and depression is controversial. The third National Health and Nutrition Examination Survey (NAHANS III) database revealed that the association between depression and elevated levels of CRP was apparent in young male adults, and the comparable association was quite weak and not significant in women [27,28]. In the current study, we did not find an association between pro-inflammatory and depressive symptoms; instead, lower IL-10, an anti-inflammatory cytokine, along with higher serotonin were the predictors for depressive symptoms in these college women. This could be because none of the study participants were depressed patients; however, the finding is echoing the recent studies that increased IL-10 can reverse depressive symptoms [29,30]. Most of the literature documented an association between a low serotonin level and depressive symptoms; however, our study found that a high level of serotonin predicted depressive symptoms. This could be due to the fact that the majority of the study participants were sleep deprived. Serotonin catalyzes the second step in melatonin formation during night or dark, when an individual did not sleep well could cause serotonin accumulated because it could not convert to melatonin [31]. Although another study also found an association between high serotonin level and depression [32], this association needs to be further explored. In addition, the complex associations between depressive symptoms, neurotransmitters (e.g., norepinephrine and dopamine), and sleep should also be examined [33].

Prior studies also found that IL-6, TNF-α, and CRP are associated with cardiovascular diseases [34,35,36,37]. In this study, none of the biomarkers were a significant predictor for the total physical symptoms; however, poor sleep quality and delayed activity rhythms were the predictors for more physical symptoms. Physical activity has been associated with depression due to neurobiological effects from noradrenergic and serotonergic systems [38]. Circadian activity rhythms have been found to be related to symptom severity in dementia patients [39], seasonal affective disorder patients [40], and cancer patients [41,42]. Circadian rhythms are generated by a central pacemaker, the suprachiasmatic nuclei (SCN), that is highly regulated and synchronized to elements of the external environment such as social activities and are closely linked to sleep, wakefulness, and health [43]. Findings from the current study warrant further study to explore the role of circadian activity rhythms in physical health among healthy women.

An unexpected methodological finding in this study was the associations among PSQI, sleep deviation index (SDI), and circadian activity rhythms (CAR). The positive correlation between PSQI and SDI (r = 0.41, *p* = 0.017) indicates SDI could be a simple and valuable indicator for clinical sleep assessment. There was a negative correlation between SDI and CAR (r = −0.34), although it was not statistically significant in this small sample, which also indicates both SDI and CAR variables should be further explored in future studies.

Although the findings from current study contribute to the knowledge in sleep disturbances and health outcomes among the general population; however, findings should be considered in light of the small sample size and a non-specific blood sample collection time point. The unexpected result of PNI biomarkers between the poor sleepers and good sleepers might be due to the small sample size of this study and the participants being healthy adults; thus, a larger scale study is needed to further validate the association between sleep disturbances and PNI biomarkers. In addition, the production of the selected biomarkers is related to circadian rhythms; however, we did not collect the blood sample in a specific time point. Therefore, a specific time point should be used to collect blood sample to increase the validity of the findings. However, an alternative explanation could be because there might be other markers which could mediate the function of the selected markers in the current study. For example, we did not examine the balance between pro-inflammatory and anti-inflammatory; thus, IL-1ra and IL-4 should be included in future studies because IL-4 is the inhibitor for IL-6, and IL-1ra (IL-1 receptor antagonist) is the inhibitor for IL-1β [3,4]. Further study should also consider the mediators of the PNI biomarkers.

## 5. Conclusions

Poor sleep quality measured by the PSQI was the significant predictor for both depressive and physical symptoms, and the poor sleepers reported significantly more depressive and physical symptoms than the good sleepers. No statistical significance was found in the selected PNI biomarkers between the poor sleepers and good sleepers; however, higher serotonin levels and lower IL-10 levels were the significant predictors for depressive symptoms. Findings also showed circadian activity rhythms play a role in the individual’s well-being since delayed peak activity rhythms is the significant predictor for physical symptoms.

The mission of NIH is to seek fundamental knowledge to enhance health, lengthen life, and reduce illness and disability, which emphasizes preventive care [44]. However, to date, the majority of studies regarding health outcomes are disease-oriented instead of using the preventive perspective by studying a healthy population. To use the general population could decrease the confounding impacts associated with the illness and provide better understanding the dynamics of sleep loss on molecular signaling pathways. A modest amount of sleep loss activates cellular markers of inflammatory responses, which are associated with up-regulation of molecular pathways that affects message abundance of inflammatory signaling pathways [45].

It has been reported that the risk factors for sleep problems are associated with older age, less education, smoking, being underweight, morbidity, and people racially identified as Black [46]. In the current study, the participants were well-educated, healthy young adults, with an average normal BMI; however, they were sleep deprived and with depressive and physical symptoms. Sleep deprivation affects performance in decreased reaction times, less vigilance, an increase in perceptual and cognitive distortions, and changes in affect [47,48]. More research is needed to further explore the risk factors for sleep disturbances and to guide interventions for this specific healthy population to improve their health status. However, college curriculum should include educational materials in the areas of sleep hygiene, associations between sleep disturbances and adverse health outcomes, and strategies to maintain synchronized circadian rhythms are needed for college women.
